# The Effect of a Hydroxytyrosol-Rich, Olive-Derived Phytocomplex on Aerobic Exercise and Acute Recovery

**DOI:** 10.3390/nu15020421

**Published:** 2023-01-13

**Authors:** Justin D. Roberts, Joseph B. Lillis, Jorge Marques Pinto, Havovi Chichger, Álvaro López-Samanes, Juan Del Coso, Rodrigo Zacca, Ashley G. B. Willmott

**Affiliations:** 1 Cambridge Centre for Sport and Exercise Sciences (CCSES), School of Psychology and Sport Science, Anglia Ruskin University, Cambridge CB1 1PT, UK; 2 School of Life Sciences, Anglia Ruskin University, Cambridge CB1 1PT, UK; 3 Exercise Physiology Group, Faculty of Health Sciences, Universidad Francisco de Vitoria, 28223 Madrid, Spain; 4 Centre for Sport Studies, Rey Juan Carlos University, 28943 Fuenlabrada, Spain; 5 Research Center in Physical Activity, Health and Leisure (CIAFEL), Faculty of Sports, University of Porto (FADEUP), 4200-450 Porto, Portugal; 6 Laboratory for Integrative and Translational Research in Population Health (ITR), 4050-600 Porto, Portugal

**Keywords:** polyphenols, OliPhenolia^®^, hydroxytyrosol, exercise, oxygen uptake kinetics, lactate threshold, running economy

## Abstract

There is current scientific interest in naturally sourced phenolic compounds and their potential benefits to health, as well as the effective role polyphenols may provide in an exercise setting. This study investigated the chronic effects of supplementation with a biodynamic and organic olive fruit water phytocomplex (OliPhenolia^®^ [OliP]), rich in hydroxytyrosol (HT), on submaximal and exhaustive exercise performance and respiratory markers of recovery. Twenty-nine recreationally active participants (42 ± 2 yrs; 71.1 ± 2.1 kg; 1.76 ± 0.02 m) consumed 2 × 28 mL∙d^−1^ of OliP or a taste- and appearance-matched placebo (PL) over 16 consecutive days. Participants completed a demanding, aerobic exercise protocol at ~75% maximal oxygen uptake ($\dot{V}$O_2max_) for 65 min 24 h before sub- and maximal performance exercise tests prior to and following the 16-day consumption period. OliP reduced the time constant (τ) (*p* = 0.005) at the onset of exercise, running economy (*p =* 0.015) at lactate threshold 1 (LT1), as well as the rating of perceived exertion (*p* = 0.003) at lactate turnpoint (LT2). Additionally, OliP led to modest improvements in acute recovery based upon a shorter time to achieve 50% of the end of exercise $\dot{V}$O_2_ value (*p* = 0.02). Whilst OliP increased time to exhaustion (+4.1 ± 1.8%), this was not significantly different to PL (*p* > 0.05). Phenolic compounds present in OliP, including HT and related metabolites, may provide benefits for aerobic exercise and acute recovery in recreationally active individuals. Further research is needed to determine whether dose-response or adjunct use of OliP alongside longer-term training programs can further modulate exercise-associated adaptations in recreationally active individuals, or indeed support athletic performance.

## 1. Introduction

Nutritional strategies to enhance exercise performance and recovery are of current scientific interest to individuals who regularly undertake physical activity, competitive athletes, military workers, as well as the general population. Recent approaches which have gained popularity in an attempt to attenuate exercise-induced muscle damage (EIMD) and oxidative stress include the supplementation of naturally occurring phytochemicals (i.e., polyphenols) from sources such as pomegranate, cocoa, or cherries [1,2,3]. The average adult consumption of polyphenols is suggested to be ~1 g·d^−1^ [4], with primary sources from fruits, vegetables, beverages such as tea and coffee, wine, and chocolate [5]. With antioxidant properties [6], nutritional polyphenols may act as radical scavengers and metal chelators, regulating metabolism, body mass, chronic disease, and cell proliferation [7]. Free radicals and reactive oxygen and nitrogen species (RONS) are the primary oxidizing agents produced in cellular biochemical reactions for aerobic energy production [5]. Aerobic exercise is characterized by increased total energy expenditure [8], where the availability of endogenous substrates and aerobic metabolism are crucial for overall performance [9,10]. The increased oxygen (O_2_) demand by skeletal muscles during exercise results in greater free radical production and an increase in RONS [11]. Whilst viewed as detrimental to the cell for many years, recent evidence shows that RONS are crucial physiological activators and regulators of various intracellular signaling pathways in response to stress, enhancing defense, improving cell adaptation, and upregulating the expression of endogenous antioxidant enzymes [12,13].

Furthermore, exercise adaptations are dependent, at least partially, on an acute oxidative stress response. When exercise intensity is matched, individuals expressing lower levels of RONS have demonstrated inferior training adaptations compared to those with moderate or higher levels of RONS [14]. However, during excessive and demanding exercise, an imbalance between RONS and endogenous antioxidants induces oxidative damage, potentially impacting at a mitochondrial or DNA level [15], reducing vasodilatory capacity [16] and contractile force within the muscle through impaired calcium sensitivity [17]. This can have inferences for repetitive training sessions or longer-term adaptations and may, therefore, impair exercise performance and/or the recovery process. In sports where arterial blood flow and maximum cardiac output are determinants of performance (i.e., endurance and team-based sports), acute ingestion (<3 h before competition) or chronic supplementation of polyphenols (~7-days) could improve time to exhaustion at 70% maximum oxygen uptake ($\dot{V}$O_2max_) by +9.7% [18] and intermittent high-intensity running distance by +10% [19]. 

The mechanisms by which polyphenols may facilitate ergogenic effects reportedly occur via nitric oxide synthase production [20] as well as the activation of sirtuin 1 (SIRT1) [21,22]. SIRT1 deacetylates several transcription factors such as forkhead (FOXO) proteins and peroxisome proliferator-activated receptor gamma coactivator 1-alpha (PGC-1α) [23]. This can facilitate mitochondrial biogenesis, endothelial function, cell proliferation and differentiation, metabolic efficiency, resistance to stress, and improve inflammatory and immune function [24,25,26]. The supplementation of phenolic compounds, and gut-derived metabolites, may therefore provide adjunct or indirect ergogenic effects on physical performance by way of potentially reducing the O_2_ cost of exercise (i.e., economy), enhancing $\dot{V}$O_2max_ or exercise tolerance, and/or improving substrate utilization efficiency. Previous findings have highlighted that polyphenol nutrients (e.g., resveratrol) may support mitochondrial function [27] and may therefore modulate essential biological functions (including thermogenesis, mitochondrial biogenesis and adenosine triphosphate production) [28]. These functions are pivotal for trained, recreationally active and untrained exercising individuals, ensuring that substrate supply kinetics and waste product removal match the requirements of the specific exercise bout [29].

Furthermore, it could be inferred that due to the anti-inflammatory and immuno-modulatory effects of phenolic compounds, an increase in polyphenol consumption (from food or supplementation) may be pertinent to exercise recovery. A reduction in physiological stressors that negatively impact exercise training [30] may support fast and slow phases of recovery, influencing performance in both prolonged or repeated bouts of exercise. Evidence for enhanced functional recovery from foods/supplements high in polyphenol compounds (e.g., Montmorency cherries > 5-days) have been exhibited in both trained and untrained individuals in a multitude of general exercise settings [31,32,33]. However, further research is warranted to investigate other polyphenols or novel food products, to assess markers of exercise recovery and identify the potential impact of phenolic compounds in specific exercise settings.

This is the first study to undertake an investigation into a commercially available polyphenol-rich olive fruit water, OliPhenolia^®^ (OliP), which has not been assessed in an exercise domain. Originating during the olive picking season, this polyphenol-rich drink is extracted via concentration, reverse osmosis, and ceramic membrane technology at the aqueous part of the olive fruit. Whilst OliP contains a variety of phenolic compounds, it is particularly rich in hydroxytyrosol (HT). Abundant in olives in the form of pure HT, HT glycosides and oleuropein, HT is an effective antioxidant, with studies highlighting protection against oxidative stress in vascular tissue [34,35], low-density lipoprotein oxidation [36,37,38], and a reduction in oxidative damage in intestinal epithelial cells [39], hepatocytes, and erythrocytes [40]. However, OliP has yet to be considered within an exercise and/or recovery domain and thus, requires investigation. Therefore, this study investigated the effect of OliP on submaximal and exhaustive exercise, as well as respiratory markers of acute recovery, in recreationally active volunteers. Understanding the efficacy of OliP may inform future nutritional strategies pertinent to exercise training and recovery.

## 2. Materials and Methods

### 2.1. Ethical Approval and Trial Registration

This study was registered with clinical-trials.gov (ID: NCT04959006) with ethical approval obtained from the Faculty of Science and Engineering Research Ethics Panel, Anglia Ruskin University (Ethical approval no. FSE/FREP/20/946). Following a priori power calculation (G*power3, Dusseldorf, Germany [41]) using ɑ = 0.05 and 1-β = 0.80, from previous reports of a time trial run and following recovery (plasma free radicals, post run pain and time to recovery [h]) [42], a minimum sample size of eight per intervention group was estimated. 

### 2.2. Participant Characteristics

Eligibility for the study required participants to be recreationally active (undertaking ~3 exercise sessions a week), with a $\dot{V}$O_2max_ of >25 mL·kg^−1^·min^−1^ determined at the first visit. All participants were >21 yrs, with no known metabolic disorders, viruses, or infections; were not self-administering any polyphenol or antioxidant-rich supplementation or adhering to specific diets that could conflict with study parameters. A total of 32 healthy participants volunteered and engaged with the study. However, following a review of individual protocol adherence and analysis of outliers, 3 participants’ datasets were removed. General characteristics of the remaining 29 participants satisfactorily completing the study are displayed in Table 1.

### 2.3. Experimental Design

Using a randomized number generator process (www.randomizer.org; accessed on 10 May 2021), participants were allocated into two supplement intervention groups (OliP or PL) in a double-blind manner. All participants reported to the Cambridge Centre for Sport and Exercise Sciences (CCSES), Anglia Ruskin University, on five separate occasions, the first of which involved an initial familiarization session [43,44]. All laboratory visits were conducted at the same time of day following an overnight fast (~10 h), with participants arriving in a euhydrated state. Participants were instructed to avoid strenuous and/or excessive exercise for the 24 h prior to testing visits, as well as adhere to all dietary instructions for the 3-days pre-testing (Section 2.4*).* For the duration of the supplementation period, participants were asked to continue habitual exercise and diet regimes, ensuring each week was matched to the previous in terms of training load, caloric and macronutrient intake.

### 2.4. Dietary and Exercise Activity Monitoring

Dietary and hydration intake was tracked via the use of a mobile based application (MyFitnessPal, Inc., San Francisco, CA, USA). Participants were provided with a personal login and guidance instructions to support detailed dietary tracking. Participants were required to record consumption of all food items and liquids for 3-days leading into each exercise test as part of this study, as well as across the 16-day intervention period [45] and were checked regularly by the same researcher for consistency throughout the intervention. A list of polyphenol-rich ‘foods to avoid’ was also provided for participants to adhere to in the 3-days leading into each laboratory visit (see Supplementary Materials, Table S1). Participants were also required to complete a standardized, daily exercise activity diary for the 3-days prior to exercise trials and the duration of the 16 consecutive days supplementation period, ensuring they were rested for the 24 h prior to each visit. Participants were requested to maintain habitual lifestyle and exercise patterns across the study, ensuring consistency across the 16-day period throughout the course of supplementation. Session type, mean session heart rate, exercise duration and perceived session exertion (using a standard 0–10 visual analogue scale) were recorded following the completion of each training session as reported elsewhere [45]. 

### 2.5. Laboratory Procedures

All tests took place under controlled environmental conditions (temperature: 19.6 ± 0.3 °C; barometric pressure: 1005.6 ± 1.2 mBar; and relative humidity: 48.4 ± 2.2%). Upon arrival, participants rested for 10 min in a seated position before assessment of blood pressure (Omron 750CP, Kyoto, Japan), body mass (electronic scale, Seca, Hamburg, Germany), and height (Seca stadiometer, Hamburg, Germany). At rest (baseline) and throughout exercise, 20 µL capilliarized fingertip blood samples were collected for the assessment of blood lactate and glucose (Biosen C Line EKF-diagnostic analyzer, Cardiff, UK). Heart rate (HR) data were recorded in 5 s intervals using a short-range telemetric monitor (Polar 810s, Polar T34 strap, Kempele, Finland). For the initial familiarization trial, body composition was also recorded using bioelectrical impedance for the indirect assessment of body fat percentage, fat-free mass, and fat mass (Tanita SC-330ST, Amsterdam, The Netherlands). Breath-by-breath pulmonary gas variables (volume of O_2_ [$\dot{V}$O_2_], volume of carbon dioxide [$\dot{V}$CO_2_], minute ventilation [$\dot{V}$_E_], respiratory exchange ratio [RER], breathing frequency [BF] and tidal volume [TV]) were measured continuously via a metabolic cart (MetaLyzer 3B-R2, Cortex Ltd., Leipzig, Germany) using a suitable facemask for each participant (7600 face mask with headgear, Hans Rudolph, Shawnee, Kansas, USA). Prior to each test, the MetaLyzer was calibrated as per manufacturers’ specifications. All exercise testing was completed on a Quasar Med Treadmill (HP Cosmos, Nussdorf, Germany).

#### 2.5.1. Experimental Protocols—Visit 1, 3 and 5

**Figure 1:**
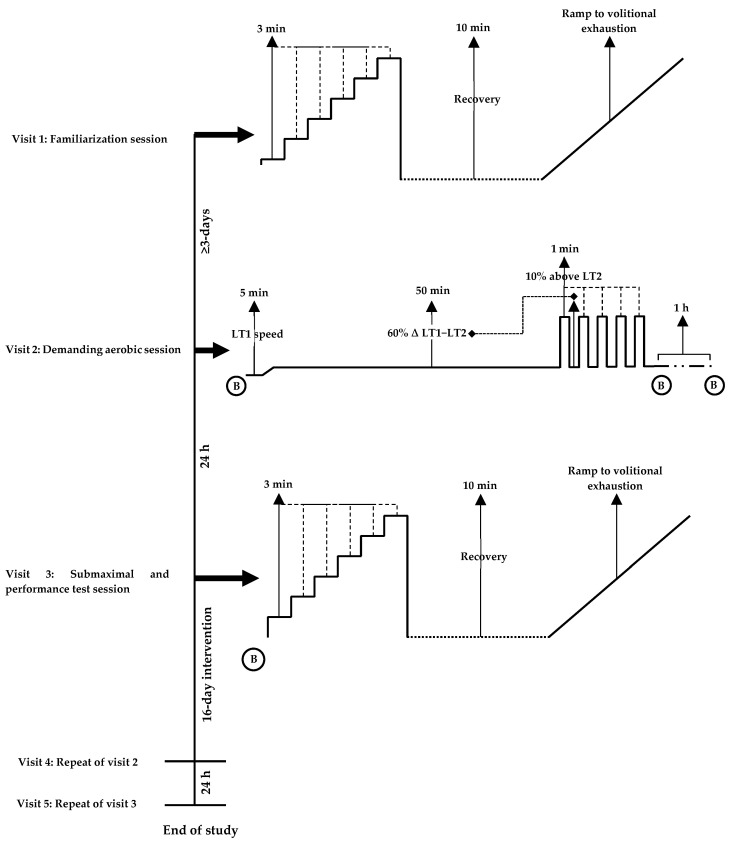
Schematic of study protocol outlining the familiarization (visit 1), demanding aerobic session (visit 2) and submaximal and performance test session (visit 3). B = blood sample; LT1 = lactate threshold; LT2 = lactate turnpoint.

Exercise intensities were calculated using lactate profiles from the familiarization trial (visit 1) and remained consistent in visit 3 and 5. Visits 1, 3 and 5 consisted of a two-part graded exercise test [46,47] including: (1) a submaximal incremental protocol, with a 10 min recovery period; and (2) a maximal test to volitional exhaustion (Figure 1).

#### 2.5.2. Submaximal and Performance Test Protocol

The speed for the submaximal protocol was selected at a pre-defined level and increased by 1 km∙h^−1^ every 4 min, with 3 min of running at a constant speed [46,47] followed by a 1 min break for capilliarized fingertip blood sample collection. The gradient was maintained at 1% [48] with rating of perceived exertion (RPE; 0−10 scale) and HR assessed in the final 30 s of each running stage. For the $\dot{V}$O_2max_ performance test, speed was held consistent with gradient increasing by 1% per min, with RPE and blood lactate concentration (B[La]) obtained at the end of the test. Participants ran until volitional exhaustion (which determined time to exhaustion [TTE]), with standardized verbal encouragement provided towards the end of the test.

#### 2.5.3. Determination of Physiological Parameters and Respiratory Kinetics

Lactate threshold (LT1) was determined by an initial rise in B[La] above baseline [49], and lactate turnpoint (LT2) was determined by a sudden and sustained increase in B[La] [50]. Mean and standard deviations (SD) of $\dot{V}$O_2_ for the last 30 breaths of each increment were calculated. Values ±4 SD were removed as outliers with all remaining breaths averaged [44].

Running economy was calculated in mL·kg^−1^·km^−1^ [51], described in Equation (1) below:

(1) $$\mathrm{Economy}=\frac{{\dot{V}O}_{2}\left({\mathrm{mL}\cdot \mathrm{kg}}^{-1}\cdot \min^{-1}\right)}{{\mathrm{Speed}\;(\mathrm{km}\cdot h}^{-1})/60}$$

The $\dot{V}$O_2_ kinetics for exercise (on-kinetics) and recovery periods (off-kinetics) were modelled and calculated using validated software VO_2_FITTING [52]. Errant breaths were omitted by only including those within $\dot{V}$O_2_ local mean ± 4 SD. Subsequently, the individual on-transient breath-by-breath $\dot{V}$O_2_ responses were modelled using a mono-exponential model [52] described in Equation (2):

(2) $${\dot{V}O}_{2}\left(t\right)={\dot{V}O}_{2\mathrm{baseline}}+A\left(1-e^{-}{}^{\frac{t}{\tau }}\right)$$

where $\dot{V}$O_2_(*t*) represents the relative $\dot{V}$O_2_ at the time *t*, A and τ are the amplitude and time constant (τ) of the fast $\dot{V}$O_2_ component. The individual off-transient breath-by-breath $\dot{V}$O_2_ responses were modelled using the a mono-exponential model [52] described in Equation (3):

(3) $${\dot{V}O}_{2}\left(t\right)={\mathrm{EE}\dot{V}O}_{2}-A\left(1-e^{-}{}^{\frac{t}{\tau }}\right)$$

where EE$\dot{V}$O_2_ represents the relative end-exercise $\dot{V}$O_2_ during the on-transient kinetics phase. During exercise (on-kinetics), O_2_ deficit, $\dot{V}$O_2_ demand and τ were estimated [53]. The acute recovery period in this study reflected the 10 min following the submaximal exercise protocol. Within this period, time to 50% (T50%) was determined by the recording of consistent breaths under 50% of the $\dot{V}$O_2max_ value reached [19]. $\dot{V}$O_2max_ was determined from the highest $\dot{V}$O_2_ values recorded over a 15-breath rolling average [54]. 

#### 2.5.4. Demanding Aerobic Session—Visit 2 and 4

Visit 2 and 4 involved 65 min of exercise, with an overall target exercise intensity of ~75% $\dot{V}$O_2max_, designed to elicit muscular oxidative stress [45]. Participants completed a 5 min warm-up at a speed corresponding to LT1. Exercise intensity then increased to speeds that corresponded with 60% of the difference between LT1 and LT2 (∆LT1-LT2) for 50 min, before completing a maximum of 5 × 1 min intervals at a speed 10% above LT2, interspersed with 1 min active recovery at 60% ∆LT1-LT2. B[La], HR and RPE were measured at rest, and at 10, 30 and 48 min, and following the last interval. Exercise intensity was consistent between visit 2 and 4. Additionally, as a means to quantify whether the nutritional intervention influenced plasma HT (as the main polyphenol in OliP), resting whole blood measures were undertaken prior to both visit 2 and 4 (as part of a larger study reported elsewhere [45]). For this, whole blood samples were collected into 4 mL Vacuette™ K2EDTA tubes (Greiner Bio-One GmbH, Kremsmunster, Austria), centrifuged at 2000 rcf for 10 min, with extracted plasma stored at −80 °C until analysis for HT. Plasma HT was assessed using a liquid–liquid extraction method following acidic hydrolysis, with gas chromatography–mass spectrometry (GC-MS) analysis (Agilent 7820A GC, Santa Clara, CA, USA [45]). 

### 2.6. Nutritional Intervention

Nutritional supplementation was distributed in a double-blinded manner to participants upon completion of visit 3. Product dose and timeframe were based on commercial product supply and company recommendations to consume 1 box (32 jars) of OliP as an acute intervention period. Therefore, participants were provided 32 jars in identically labelled boxes and requested to consume 2 jars per day (56 mL total) separated by ~6 h between meals across 16-days. Each jar contained ~28 mL of either OliP (sweetened version, Batch 14, Fattoria La Vialla, Castiglion Fibocchi, Arezzo, Italy [see Supplementary Materials, Table S2 for independent product analysis]) or taste- and appearance-matched PL (equal ratio of: prune juice [Sunsweet California Prune Juice, Tesco, Welwyn Garden City, UK], diet cola [Tesco Cola, Tesco, Welwyn Garden City, UK] and tonic water [low-calorie Indian tonic water, Tesco, Welwyn Garden City, UK]). To monitor adherence, participants returned all jars at the end of the trial, including any remaining full jars for confirmation of intervention adherence.

### 2.7. Dietary and Exercise Activity Analysis

Dietary analysis occurred via Nutritics Professional Dietary Analysis software (Nutritics Ltd., Co., Dublin, Ireland). Three-day dietary intake was analyzed prior to each laboratory visit to ensure study guideline protocols were adhered to (Table 2). Estimation of dietary HT intake was also assessed (excluding supplementation) using the US Department of Agriculture and the Phenol-Explorer databases. Exercise data allowed for assessment of training load, monotony and strain as previously reported [45,55] (Table 3) to quantify relative consistency between cohorts. 

### 2.8. Statistical Analysis

Statistical analysis was performed using SPSS (V.28, IBM Corporation, Armonk, New York, USA), with statistical significance determined as *p* ≤ 0.05. All data were assessed for homogeneity using Levene’s test and normality through a Shapiro-Wilk’s test [56]. A two-way repeated measures ANOVA was used for the main analysis with Greenhouse-Geisser corrections applied if sphericity could not be assumed. For plasma HT analysis, a mixed design ANOVA was also undertaken. Bonferroni post-hoc comparisons were employed where applicable, with effect sizes (partial eta squared η_p_^2^) also reported (small = 0.02, medium = 0.13, large = 0.26). An independent samples t-test was also adopted to compare relevant data between groups (i.e., participant characteristics, dietary intake and training records), whereby Cohen’s d effect sizes were utilized (trivial ≤ 0.19, small = 0.20–0.49, medium = 0.50–0.70, large ≥ 0.80). Data are presented as mean ± SE.

## 3. Results

### 3.1. Dietary Intake, Supplement Adherence, and Exercise Monitoring

No significant differences were reported within or between groups in the 3-day period leading into exercise testing for energy (kcal·d^−1^), carbohydrate (CHO), fat (FAT), and protein (PRO) intake (Table 2). HT intake during the 3-day dietary control periods prior to each visit demonstrated no differences within or between groups (*p* > 0.05). Mean intake of dietary HT during control periods prior to visit 2 and 4 were 0.06 ± 0.11 and 0.04 ± 0.10 mg for OliP, and 0.08 ± 0.13 and 0.13 ± 0.16 mg for PL respectively. Estimation of general dietary HT throughout the intervention period (excluding OliP intake) indicated a mean total intake of 0.14 ± 0.07 mg·kg^−1^·d^−1^ for OliP compared to 0.18 ± 0.11 mg·kg^−1^·d^−1^ for PL (*p* > 0.05) and was considered low. Within groups, supplementation adherence rates were 99.0% OliP and 98.1% PL, with no between group differences reported (*p >* 0.05). 

No differences were reported within or between groups during the 3-day control period for training load, monotony, or strain (*p* > 0.05, Table 3). Additionally, no differences were reported between groups for habitual exercise activity throughout the 16-day intervention (*p* > 0.05; [45]). 

### 3.2. Demanding Aerobic Session—Visit 2 and 4

No differences within or between groups were found for the aerobic test (*p* > 0.05, Table 4) demonstrating relative consistency. Overall, mean target exercise intensity of ~75% $\dot{V}$O_2max_ was achieved and sustained in both exercise sessions (76.08 ± 1.04 and 75.42 ± 0.98%, visit 2 and 4, respectively). Exercise intensity was consistent between groups for visit 2 and 4 in the OliP (11.4 ± 1.7 km·h^−1^) and PL group (11.5 ± 1.8 km·h^−1^; both *p* > 0.05). Plasma HT was not detected at baseline (visit 2, pre-supplementation) or following PL, but significantly increased from 0.0 ± 0.0 to 6.3 ± 1.6 ng·mL^−1^ following OliP (F = 14.28, *p* = 0.001, η_p_^2^ = 0.43).

### 3.3. Submaximal Exercise—Visit 3 and 5

**Figure 2:**
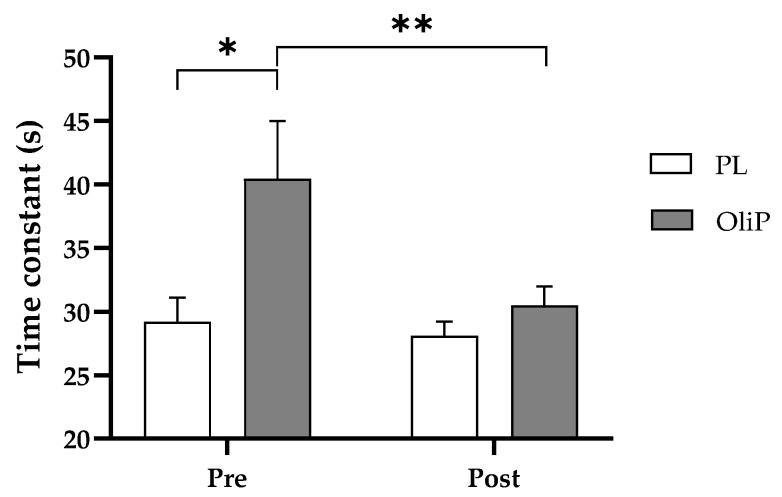
Time constant (τ) at the onset of exercise pre- and post-intervention for OliP and PL groups. * denotes significant difference between groups pre intervention (*p* = 0.047), ** denotes significant difference between time points for OliP (*p* = 0.005).

Onset of exercise: For time constant (τ), there was a significant effect for time (F = 5.23, *p* = 0.031, η_p_^2^ = 0.17) and group (F = 4.44, *p* = 0.045, η_p_^2^ = 0.15). A significant difference between groups pre-intervention (visit 3) was found (F = 4.36, *p* = 0.047, η_p_^2^ = 0.15). A significant reduction from visit 3 and 5 was found in τ within OliP (F = 9.51, *p* = 0.005, η_p_^2^ = 0.28, Figure 2, Table 5A) only. No differences were found for the PL group (*p* > 0.05).

**Figure 3:**
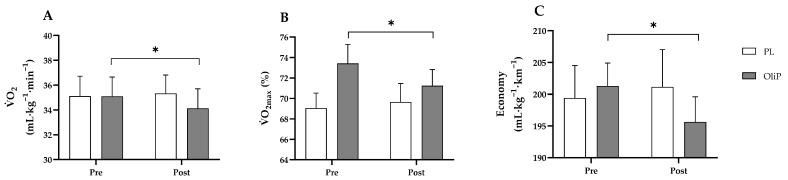
Summary respiratory and economy differences at lactate threshold (LT1) intensity pre and post 16 consecutive days consumption of either OliP or PL for (**A**) $\dot{V}$O_2_; (**B**) $\dot{V}$O_2max_ % of baseline and (**C**) running economy. * denotes significance between time points in the OliP group (*p* < 0.05).

Lactate threshold 1 (LT1): Respiratory parameters, exercise economy, B[La] and RPE are shown in Table 5A pre- to post-intervention for both OliP and PL. There was a significant interaction effect for relative $\dot{V}$O_2_ (time x group: F = 4.66, *p* = 0.039, η_p_^2^ = 0.16, Figure 3A), where a reduction in relative $\dot{V}$O_2_ was found in the OliP group between visit 3 and 5 (F = 7.09, *p* = 0.013, η_p_^2^ = 0.22). Whilst no differences were found post intervention between OliP and PL, when expressed as relative change, OliP demonstrated a −2.7 ± 1.2% reduction in $\dot{V}$O_2_ compared with the PL group at LT1 intensity (−0.7 ± 1.0%; t = 2.13, *p* = 0.043, d = 0.82, 95% confidence interval [CI] range 0.05 to 1.64). This corresponded with a significant reduction in the % of $\dot{V}$O_2max_ from baseline (73.7 ± 1.8% in visit 3 to 71.2 ±1.6% in visit 5) (F = 7.72, *p* = 0.01, η_p_^2^ = 0.24, Figure 3B) for OliP only. No differences were reported in the PL group (*p* > 0.05), or post intervention in comparison to OliP. A significant interaction effect was also observed for running economy (time x group: F = 5.22, *p* = 0.031, η_p_^2^ = 0.17, Figure 3C), with a significant improvement demonstrated between visit 3 and 5 for OliP only (F = 6.82, *p* = 0.015, η_p_^2^ = 0.21, 95% CI range 189.80 to 207.11). Finally, it was also noted that when expressed as relative change, there was a pre- to post-intervention reduction in $\dot{V}$CO_2_ within OliP (−1.6 ± 0.9%) compared to PL (+1.5 ± 0.9%; t = 2.33, *p* = 0.028, d = 0.90).

Lactate turnpoint (LT2): There was a significant interaction reported for RPE at LT2 (time x group: F = 7.99, *p* = 0.009, η_p_^2^ = 0.24), where a reduction in RPE was found in the OliP group between visit 3 and 5 only (F = 11.01, *p* = 0.003, η_p_^2^ = 0.30). No other differences were found within or between groups at this exercise intensity (*p* > 0.05, Table 5B).

### 3.4. Recovery from Submaximal Exercise

**Figure 4:**
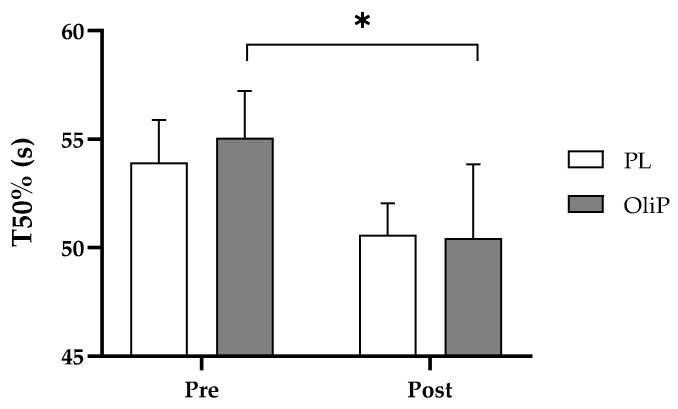
Recovery to T50% at pre- and post-intervention time points for the OliP and PL groups. * denotes significance between time points in the OliP group (*p* = 0.026).

There was a significant interaction effect for T50% in acute recovery responses (time × group: F = 7.72, *p* = 0.010, η_p_^2^ = 0.24), where post-hoc assessment indicated a reduction in T50% for the OliP group only between visit 3 and 5 (F = 5.67, *p* = 0.026, η_p_^2^ = 0.19, Figure 4). No other changes were observed for respiratory variables assessed (Table 6).

### 3.5. Time to Exhaustion and $\dot{V}$O_2max_

A significant effect was found for time during TTE (F = 11.49, *p* = 0.002, η_p_^2^ = 0.32) which increased post-intervention for both OliP (+4.1 ± 1.8%) and PL (+5.8 ± 2.6%), with no differences reported between groups for final run speed (12.6 ± 0.5 km∙h^−**1**^ for OliP, 12.9 ± 0.7 km∙h^−**1**^ for PL, *p* > 0.05, Table 7). A significant interaction effect was reported for $\dot{V}$O_2max_ (time x group: F = 16.79, *p* = 0.033, η_p_^2^ = 0.17), where $\dot{V}$O_2max_ increased post-intervention for PL (F = 7.17, *p* = 0.013, η_p_^2^ = 0.22, 95% CI range 44.37 to 55.24), but not OliP (F = 0.16, *p* = 0.693, η_p_^2^ = 0.01, 95% CI range 347.39 to 414.41). A significant interaction effect was reported for $\dot{V}$CO_2max_ (time x group: F = 18.69, *p* = 0.018, η_p_^2^ = 0.20), with a post-intervention increase in $\dot{V}$CO_2max_ reported for the PL (F = 13.77, *p* = 0.001, η_p_^2^ = 0.36, 95% CI range 47.56 to 60.76) but not the OliP group (F = 0.61, *p* = 0.444, η_p_^2^ = 0.24, 95% CI range 47.74 to 59.54). 

## 4. Discussion

To the authors’ knowledge, this is the first study to undertake research focusing on OliP in an exercise domain and aligns with concurrent research pertinent to olive-derived phytonutrients [45]. The key findings from this study demonstrate that 16 consecutive days consumption of OliP resulted in positive effects on several key markers of running performance. Of particular interest, OliP consumption significantly improved respiratory parameters at the onset of exercise within condition (i.e., τ), and oxygen consumption and running economy at LT1 (particularly when expressed as relative change in comparison to PL). Whilst respiratory parameters at LT2 were largely unaffected by OliP, perceived exertion was improved with the phytocomplex beverage. Acute recovery (T50%) following incremental exercise was also notably improved with OliP. Whilst maximal effort and TTE measures were not different between OliP and PL, an elevated $\dot{V}$CO_2max_ was reported for PL only. Furthermore, it was noted that both groups improved TTE following the intervention. Importantly, no adverse effects were reported throughout the intervention.

Regarding methodological approaches to the demanding aerobic session, steady-state moderate intensity exercise (60–70% $\dot{V}$O_2max_) for 30–60 min followed by arduous (90% $\dot{V}$O_2max_) [57] or performance efforts [58] have been shown to provoke a heightened oxidative stress response and elicit peripheral fatigue. Accordingly, the demanding aerobic sessions employed in the current study resulted in an intensity of ~75% $\dot{V}$O_2max_, with no differences within or between groups. It can therefore be assumed that an equal degree of physiological strain was achieved between cohorts prior to the main performance tests. As dietary and exercise habits were maintained across the intervention, it is feasible that physiological adaptations observed, may therefore be partly attributed to the phenolic compounds within OliP. As a naturally derived phytocomplex, OliP is notably rich in HT, which is a key polyphenol of interest and may support endogenous antioxidant mechanisms pertinent to mitochondrial respiratory capacity and/or efficiency, such as upregulation of PGC-1α [28,59,60,61,62]. 

Consumption of OliP may therefore be of relevance to individuals who engage in regular aerobic exercise, considering the negligible dietary HT content in both the pre-visit control period and habitual diet assessments for both cohorts. Plasma HT concentrations were not detected at baseline (pre-supplementation), or post PL, but were significantly elevated in response to the OliP intervention. Therefore, any impact on aerobic exercise may be associated with increased systemic HT concentrations, or gut-derived metabolites. At present, however, there is a paucity of scientific research surrounding HT and exercise performance. Additionally, there does not appear to be any existing research evidencing the effects of HT on aerobic running performance in humans. Plant-based polyphenols have peaked scientific interest in recent years [29,63], in particular HT, due to its potential to impact multiple physiological pathways. In an exercise domain, recent animal studies have demonstrated the ability of HT to enhance endurance capacity [59], prevent exercise induced fatigue, muscle damage and immunosuppression [64,65] and improve mitochondrial function in both trained and sedentary rodents [65]. However, these findings need to be corroborated in human models as well as within an exercise domain to ascertain the efficacy of HT-rich supplements.

It is also important to outline the current debate surrounding the efficacy of antioxidant and polyphenol supplementation as an exercise or training aid. Adaptations from exercise are dependent, at least partially, on individual oxidative stress responses [66]. One perspective highlights the potential inhibition of natural training adaptations through limiting the upregulation of endogenous antioxidant enzymes, and therefore diminishing the hormetic response to moderate exercise [13]. However, the counterargument highlights that the subsequent reduction in oxidative stress following antioxidant and/or polyphenol supplementation may positively influence recovery kinetics, development in contractile force, calcium handling, and therefore the ability to exercise and/or recover more ‘economically’. This may facilitate adaptations to exercise training and/or athletic performance [17]. 

Findings from this study demonstrated a ~17% improvement in τ at the onset of exercise for OliP. τ reflects the speed at which the steady-state is achieved [53], and in turn the size of the O_2_ deficit [67]. However, these results were only significant within condition and should therefore be interpreted with caution. In addition, it was noted that non-significant differences were observed between conditions prior to the nutritional intervention based on random participant allocation, which may in part impact the observed findings. Contrary to these findings, Breese et al. [68] reported no differences in $\dot{V}$O_2_ phase II time constant, from unloaded to moderate exercise after 6-days supplementation with beetroot juice (BTJ; ~8 mmol nitrate (NO_3_^−^)). Although mitochondrial respiratory capacity was not assessed, it is known that the speed of the O_2_ uptake response during the onset of moderate exercise intensity is associated with the respiratory capacity of mitochondrial complex II and the capacity of the mitochondrial electron transport system [69,70]. As HT has been shown to improve the expression of mitochondrial complex I/II/IV, this is of particular interest in an exercise domain as complex I is recognized to be the primary complex for the electron transport chain [71]. Moreover, HT has been reported to promote the congregation of complex I (CI) into supercomplexes (SCs) [65], therefore decreasing the diffusion distance for transfer of electrons between complexes, and improving the efficiency of the mitochondrial electron transfer between complexes [59,72]. More research is required to ascertain the above stated mechanisms in humans, particularly in relation to OliP consumption. 

Consumption of OliP in the dose provided also resulted a significant decrease (−2.7%) in $\dot{V}$O_2_ consumption at LT1 compared with PL. This aligns with existing research into both high [73]- and low [74]-dose BTJ supplementation whereby a ~5% reduction $\dot{V}$O_2_ consumption was reported with no changes in $\dot{V}$_E,_ RER or HR [74]. This modest change could be partially attributed to the increase in mitochondrial function and increased expression of PGC-1α following supplementation of OliP. In vitro, HT administration has been shown to upregulate nuclear respiratory factors 1 and 2, mitochondrial transcription factor A, and peroxisome proliferator active receptor γ (PPAR γ) in response to increased phosphorylation of adenosine monophosphate kinase (AMPK) [61]. The role HT may play in enhancing mitochondrial respiratory capacity could also provide a rationale for the reduced oxygen consumption observed during sub-maximal exercise at low to moderate intensities (LT1). In vitro, HT has been shown to improve mitochondrial biogenesis, O_2_ and fatty acid utilization in adipocyte cells [61,75,76]. Although not measured in this study, this may support the proposed benefits of OliP in a submaximal exercise domain, however, more research is required in humans to confirm such mechanisms. It is also viable that other phenolic compounds [38] (i.e., oleuropein aglycone) and HT derivatives (i.e., HT glucosides) found in OliP may also support antioxidant pathways that may influence aerobic performance [59,77]. Indeed, olive-derived phenolic compounds are not entirely absorbed during digestion and are extensively transformed into different metabolites by the gut microbiota [78]. For instance, whilst oleuropein transformation by gut bacteria can increase HT yield [79], HT is further transformed into homovanillin derivatives [80,81] and glutathione conjugates [79] which may have pertinent antioxidant properties [82]. These metabolites may exert further physiological effects [83] potentially explaining findings from the current study. Furthermore, HT-derived metabolite variability and quantity are also dependent on the phenolic composition of the product consumed [81]. Such complexities should also be considered when determining the physiological impact of combined polyphenols.

A relevant parameter of aerobic performance is the efficiency of movement, i.e., exercise economy [84]. This reflects the amount of O_2_ required to generate a constant submaximal running speed and therefore, is directly associated with the efficiency of aerobic fuel metabolism and the sparing of glycogen reserves [85]. Mitochondria are crucial for aerobic energy generation in exercise [86]. Improvement in mitochondrial respiratory capacity and functional efficiency following HT supplementation in animal studies has been established [87] and is associated with the constitution of supramolecular entities, the mitochondrial SCs, including respiratory complex I, III, and IV [88]. Administration of HT for 10-weeks in rodents (20 vs. 300 mg·kg^−**1**^·d^−**1**^) and exercise (up to 65 min a day at 75% of maximal velocity) compared to exercise alone improved mitochondrial function and antioxidant capacity induced by exercise [65]. However, when the HT dose was increased to 300 mg·kg^−**1**^·d^−**1**^, pro-oxidant effects were evident [65], which appeared to negatively influence SCs assembly, aligning with existing published literature [64]. Collectively, these results indicate that whilst exercise induces the formation of mitochondrial SCs [89], low-dose HT consumption may support or enhance this process [65] whilst a high dose of HT may provoke pro-oxidant mechanisms, disrupting the mitochondria and potentially limiting or diminishing SC adaptation [64]. In the current study, a relatively low HT dose was employed as part of the olive-derived phytocomplex (~0.8 mg·kg^−**1**^·d^−**1**^) in healthy volunteers. Whilst mitochondrial function was not directly assessed, it is feasible that HT and related gut-derived phenolic metabolites may have supported SC assembly and facilitated improved oxygen cost responses observed at the onset of exercise and during low to moderate exercise (LT1). Furthermore, the low HT dose employed in the current study may also explain why exercise performance (TTE) was not significantly different between cohorts in line with previous research [64].

Despite OliP presenting no significant impact on respiratory mechanisms at LT2, a poignant finding was the observed significant reduction in RPE at this intensity. Mechanisms for this are unclear, however it is feasible that there may be a link to a reduction in brain oxygenation that is present during intensive exercise and directly associated with an increase of fatigue (subjectively quantified as perceived exertion) [90]. Alternatively, mechanisms potentially occurring at a mitochondrial level and the effect upon SCI and SCII, may indicate that beneficial responses to OliP are more likely to be present at lower intensities only. Further research is required to accurately ascertain potential mechanisms involved in subjective measures associated with exercise. 

Similarly, recovery was largely unaffected based upon off-kinetic modeling; however, current findings did present a −9.4% decrease in T50% for OliP compared with a −5.6% decrease in PL, during the initial recovery period from sub-maximal exercise. This in itself warrants further investigation considering that previous findings utilizing a similar exercise intensity (70% maximum aerobic power) did not find a benefit to $\dot{V}$O_2_ half-recovery time following the supplementation of mixed polyphenols (250 mg Vinitrox^™^ for 7-days [19]). In the current study it is feasible that the HT content in OliP (and related gut-dervied metabolites) may be influencing recovery indirectly, and may therefore have applications following repeated bouts of exercise. However, results should be interpreted with caution and further research should be undertaken to corroborate findings. Finally, although improvements in TTE were evident in both groups (+4.1% OliP and +5.8% PL), the overall change in exercise performance was not different between OliP and PL. In the current study, exercise performance was based upon physical tolerance to sustained near-maximal exercise. Based upon findings at LT1 intensity, it could be prudent to assess whether OliP is more effective when determining performance employing other measures such as an extended time trial (i.e., 5 km run time) or total work completed in a fixed time period as opposed to an acute near-maximal TTE bout.

### Study Limitations and Future Directions

It is important to note that there were several limitations to the current study. Firstly, improvements were found in specific, but not all parameters assessed. As example, change in time constant at the onset of exercise was noted within-group only for OliP and therefore should be interpreted with caution. Likewise, during acute recovery, whilst improvements were observed for T50%, other parameters using respiratory off-kinetics were not deemed significant, and again results should be interpreted carefully. However, where main interaction effects were identified (including relative changes in oxygen consumption and running economy at LT1 compared with PL) important adaptations following the inclusion of dietary OliP may be evident. It should, however, be noted that differences were not observed post-intervention between conditions which should be taken into consideration.

Although improvements were observed particularly at LT1 with healthy, recreationally active volunteers, we did not specifically distinguish whether such effects were pertinent to gender, training status, or the type/intensity of habitual exercise. Further research may therefore be relevant to determine the potential applications of OliP in various cohorts. Additionally, the protocol used in the current study was designed to standardize the demanding aerobic run prior to the following day exercise performance session for all participants [45]. It is important to recognize the translation of controlled laboratory findings to real-world exercise applications [91], and future research should investigate the adjunct use of OliP in applied and field-based settings (e.g., single exercise sessions, events that require repeated bouts, or multi-day events). It should also be noted that existing literature has outlined the potential variability in polyphenol products [92]. Whilst 16-days of OliP consumption (Batch 14) positively influenced aerobic exercise parameters and acute recovery, results may differ between batches and additional investigation is needed to corroborate current findings. Indeed, as previously noted, the intestinal microbiota plays an important role influencing gut-derived phenolic metabolites, which are additionally dependent on the phenolic composition of dietary products. 

As this was the first study to assess the use of OliP in an exercise domain, a parallel cohort design was employed to ascertain the influence of a single course of the phytocomplex (16 consecutive days) whilst minimizing potential for longer term training effects. Further research should investigate whether time course (>16-days), dose-response (>56 mL·d^−**1**^) and/or dose-frequency (>2 serves·d^−**1**^) can influence sustained exercise training adaptations or accumulated recovery, i.e., during marathon training. Additional exploration into alternate recovery periods (i.e., respiratory measures up to 1 h post exercise, and inflammatory or muscle soreness measures 1, 12, 24, and 48 h+ following exhaustive exercise) is also warranted. Finally, based upon current findings, including effects of OliP on exercise-induced oxidative stress presented elsewhere [45], it would be beneficial to assess the potential impact of this olive-derived phytocomplex on inflammatory markers associated with EIMD (particularly within other populations, e.g., trained athletes), or within clinical applications where functional movement may be impacted (e.g., arthritis, fibromyalgia).

## 5. Conclusions

This is the first study to investigate the use of OliP in an exercise domain. Findings demonstrated that 16-days supplementation of OliP positively influenced parameters of aerobic exercise, most notably at submaximal levels. Reduced oxygen cost and improved running economy at exercise intensities corresponding with LT1, as well as improvements in acute recovery may have implications for recreationally active individuals undertaking demanding or repeated aerobic exercise training. Further research is warranted to corroborate these findings and explore potential applications (time-course, dose-response) to prolonged training periods and/or repetitive bouts of exercise. 

## Figures and Tables

**Table 1:** Mean ± standard error (SE) participant characteristics overall and for OliPhenolia^®^ (OliP) and placebo (PL) groups respectively.

Variable	Overall	OliP	PL
	(*n* = 29; 20 M, 9 F)	(*n* = 15; 11 M, 4 F)	(*n* = 14; 9 M, 5 F)
Age (yrs)	42 ± 2	42 ± 3	42 ± 3
Height (m)	1.76 ± 0.02	1.77 ± 0.03	1.75 ± 0.03
Body mass (kg)	71.08 ± 2.14	73.57 ± 2.44	68.41 ± 3.52
Fat free mass (kg)	57.67 ± 2.31	59.33 ± 3.05	55.89 ± 3.56
Body mass index (kg·m^2^)	22.9 ± 0.4	23.5 ± 0.4	22.3 ± 0.7
Body fat (%)	18.7 ± 1.8	19.5 ± 2.2	17.8 ± 3.0
$\dot{V}$O_2max_ (L·min^−1^)	3.53 ± 0.16	3.56 ± 0.22	3.49 ± 0.24
$\dot{V}$O_2max_ (mL·kg^−1^·min^−1^)	49.6 ± 1.7	48.3 ± 2.5	51.0 ± 2.2

M = male; F = female; $\dot{V}$O_2max_ = maximal oxygen uptake. No statistical differences were reported between groups (*p* > 0.05).

**Table 2:** Energy and macronutrient intake for both groups within the 3-day control period prior to visit 2 (pre-intervention) and 4 (post-intervention).

Variable	Time	OliP	PL
Kcal·d^−1^	Pre	2134.9 ± 139.7	2149.6 ± 112.3
	Post	2172.1 ± 135.5	2456.8 ± 151.2
CHO (g·d^−1^)	Pre	232.9 ± 17.4	259.7 ± 15.9
	Post	240.0 ± 16.1	273.8 ± 22.8
CHO_relative_ (g·kg^−1^·d^−1^)	Pre	3.2 ± 0.3	3.9 ± 0.2
	Post	3.3 ± 0.2	4.1 ± 0.3
FAT (g·d^−1^)	Pre	85.9 ± 6.2	76.7 ± 4.8
	Post	87.0 ± 6.4	97.9 ± 6.2
FAT_relative_ (g·kg^−1^·d^−1^)	Pre	1.2 ± 0.1	1.2 ± 0.1
	Post	1.1 ± 0.1	1.5 ± 0.1
PRO (g·d^−1^)	Pre	103.0 ± 8.6	104.2 ± 7.6
	Post	103.4 ± 7.7	115.6 ± 9.1
PRO_relative_ (g·kg^−1^·d^−1^)	Pre	1.4 ± 0.1	1.5 ± 0.1
	Post	1.4 ± 0.1	1.7 ± 0.2

No statistical differences were reported between groups (*p* > 0.05). Units: Kcal·d^−1^ = kilocalories per day; g·d^−1^ = grams per day; g·kg^−1^·d^−1^ = grams per kilogram body mass per day.

**Table 3:** Mean habitual exercise activity for both groups within the 3-day control period prior to visit 2 (pre-intervention) and 4 (post-intervention), as well as collated mean training parameters across the intervention period.

Control Period (3-Days) Prior to Laboratory Visits			
Variable	Time	OliP	PL
Training load (AU)	Pre	641 ± 106	646 ± 116
	Post	722 ± 80	802 ± 150
Training monotony (AU)	Pre	0.9 ± 0.1	1.0 ± 0.1
	Post	1.0 ± 0.1	1.2 ± 0.1
Training strain (AU)	Pre	730 ± 182	583 ± 118
	Post	753 ± 143	1112 ± 272
**Across the 16-day intervention**			
Exercise sessions completed		13 ± 1	15 ± 1
Session duration (min)		58.6 ± 4.2	67.3 ± 4.9
HR (b∙min^−1^)		135 ± 4	140 ± 4
Session perceived exertion		5.2 ± 0.3	4.8 ± 0.3

AU = arbitrary units. No statistical differences were reported between groups (*p* > 0.05).

**Table 4:** Physiological responses for both groups during the demanding aerobic session at visit 2 (pre-intervention) and 4 (post-intervention).

Variable	OliP		PL	
	Pre	Post	Pre	Post
$\dot{V}$O_2_ (mL·kg^−1^·min^−1^)	36.6 ± 1.5	36.7 ± 1.5	37.7 ± 1.6	37.4 ± 1.4
% of baseline $\dot{V}$O_2max_ (%)	76.6 ± 1.5	76.7 ± 1.2	75.0 ± 1.8	74.6 ± 1.8
$\dot{V}$O_2_ (L·min^−1^)	2.70 ± 0.14	2.70 ± 0.15	2.56 ± 0.17	2.54 ± 0.15
$\dot{V}$CO_2_ (L·min^−1^)	2.49 ± 0.14	2.49 ± 0.13	2.36 ± 0.16	2.34 ± 0.15
$\dot{V}$_E_ (L·min^−1^)	85.12 ± 4.59	84.87 ± 4.68	80.99 ± 4.66	80.49 ± 4.11
$\dot{V}$_E_/$\dot{V}$O_2_	29.44 ± 0.46	29.33 ± 0.48	29.82 ± 1.01	30.01 ± 0.98
$\dot{V}$_E_ /$\dot{V}$CO_2_	31.88 ± 0.45	31.75 ± 0.46	32.34 ± 1.11	32.55 ± 1.11
RER	0.92 ± 0.01	0.92 ± 0.01	0.92 ± 0.01	0.92 ± 0.01
Economy (mL·kg^−1^·km^−1^)	193.6 ± 3.5	194.0 ± 3.4	198.4 ± 4.7	197.2 ± 4.0
B[La] (mmol·L^−1^)	**1.32 ± 0.11**	**1.31 ± 0.10**	**1.33 ± 0.11**	**1.33 ± 0.09**

$\dot{V}$O_2max_ = maximal oxygen uptake; $\dot{V}$O_2_ = volume of oxygen; $\dot{V}$CO_2_ = volume of carbon dioxide; $\dot{V}$_E_ = minute ventilation; RER = respiratory exchange ratio; B[La] = blood lactate concentration. No differences reported within or between groups (*p* > 0.05).

**Table 5:** Respiratory, exercise economy, perceived exertion, and blood lactate parameters at the onset of exercise and lactate threshold (A), and lactate turnpoint (B) at visit 3 (pre-intervention) and 5 (post-intervention) interspersed with 16 consecutive days of either OliP or PL.

**(A)**	**OliP**		**PL**	
**Onset of Exercise**	**Pre**	**Post**	**Pre**	**Post**
$\dot{V}$O_2_ at baseline (mL·kg^−1^·min^−1^)	8.48 ± 1.13	6.88 ± 0.55	6.43 ± 0.81	6.25 ± 0.60
$\dot{V}$O_2_ demand at 60 s (L)	1.69 ± 0.15	1.74 ± 0.12	1.80 ± 0.16	1.78 ± 0.14
$\dot{V}$O_2_ demand at 120 s (L)	3.39 ± 0.29	3.47 ± 0.23	3.60 ± 0.31	3.55 ± 0.28
$\dot{V}$O_2_ demand at 180 s (L)	4.80 ± 0.43	4.94 ± 0.32	5.08 ± 0.43	5.03 ± 0.40
O_2_ deficit (L)	0.97 ± 0.08	0.85 ± 0.07	0.84 ± 0.07	0.81 ± 0.07
τ (s)	40.5 ± 4.6	30.5 ± 1.5 *	29.2 ± 1.9	28.1 ± 1.1
Mean $\dot{V}$O_2_ in the last 60 s (mL·kg^−1^·min^−1^)	30.7 ± 1.5	30.0 ± 1.5	30.3 ± 2.2	31.1 ± 1.6
**Lactate threshold (LT1)**				
%$\dot{V}$O_2max_ of baseline (%)	73.0 ± 1.8	70.9 ± 1.6 *	68.7 ± 1.4	69.2 ± 1.6
$\dot{V}$O_2_ (L·min^−1^)	2.56 ± 0.13	2.50 ± 0.13 *	2.45 ± 0.15	2.46 ± 0.13
$\dot{V}$CO_2_ (L·min^−1^)	2.30 ± 0.12	2.26 ± 0.12	2.16 ± 0.15	2.19 ± 0.13
$\dot{V}$_E_ (L·min^−1^)	71.81 ± 3.55	71.36 ± 3.74	67.62 ± 4.09	68.56 ± 4.19
$\dot{V}$_E_/$\dot{V}$O_2_	26.17 ± 0.36	26.65 ± 0.42	25.83 ± 0.78	25.94 ± 0.88
$\dot{V}$_E_/$\dot{V}$CO_2_	29.22 ± 0.45	29.41 ± 0.44	29.30 ± 0.91	29.21 ± 1.04
RER	0.90 ± 0.01	0.91 ± 0.01	0.88 ± 0.01	0.89 ± 0.01
Economy (mL·kg^−1^·km^−1^)	201.3 ± 3.6	195.6 ± 4.0 *	199.4 ± 5.1	201.2 ± 5.9
RPE	2.9 ± 0.3	2.7 ± 0.3	3.2 ± 0.5	3.3 ± 0.1
B[La] (mmol·L^−1^)	1.32 ± 0.11	1.31 ± 0.10	1.33 ± 0.11	1.33 ± 0.09
**(B)**	**OliP**		**PL**	
**Lactate turnpoint (LT2)**	**Pre**	**Post**	**Pre**	**Post**
%$\dot{V}$O_2max_ (%)	84.1 ± 1.8	83.5 ± 1.4	81.2 ± 2.0	82.8 ± 2.1
$\dot{V}$O_2_ (L·min^−1^)	2.96 ± 0.16	2.95 ± 0.17	2.91 ± 0.21	2.96 ± 0.16
$\dot{V}$CO_2_ (L·min^−1^)	2.81 ± 0.15	2.81 ± 0.15	2.73 ± 0.20	2.79 ± 0.19
$\dot{V}$_E_ (L·min^−1^)	92.73 ± 4.87	92.28 ± 5.16	88.00 ± 5.48	89.80 ± 5.89
$\dot{V}$_E_ /$\dot{V}$O_2_	29.46 ± 0.50	29.41 ± 0.52	28.72 ± 0.98	28.60 ± 1.11
$\dot{V}$_E_ /$\dot{V}$CO_2_	31.00 ± 0.48	30.80 ± 0.47	30.69 ± 1.04	30.25 ± 1.13
RER	0.95 ± 0.01	0.95 ±0.01	0.94 ± 0.01	0.94 ± 0.01
Economy (mL·kg^−1^·km^−1^)	192.7 ± 4.5	191.1 ± 3.5	193.7 ± 4.7	197.7 ± 4.2
RPE	6.0 ± 0.34	5.4 ± 0.4 *	5.6 ± 0.4	5.4 ± 0.4
B[La] (mmol·L^−1^)	2.31 ± 0.12	2.18 ± 0.11	2.24 ± 0.12	2.22 ± 0.12

τ = time constant; $\dot{V}$O_2max_ = maximal oxygen uptake; $\dot{V}$O_2_ = volume of oxygen; $\dot{V}$CO_2_ = volume of carbon dioxide; $\dot{V}$_E_ = minute ventilation; RER = respiratory exchange ratio; RPE = rating of perceived exertion; B[La] = blood lactate concentration. * denotes a significant within group difference (*p* < 0.05).

**Table 6:** Recovery from submaximal exercise by intervention group (visit 3 and visit 5).

Variable	OliP		PL	
	Pre	Post	Pre	Post
EE$\dot{V}$O_2_ (mL·kg^−1^·min^−1^)	44.3 ± 2.1	43.8 ± 2.2	46.7 ± 2.4	47.0 ± 2.0
**%**$\dot{V}$O_2max_ (%)	92.8 ± 1.4	91.6 ± 1.8	91.6 ± 7.6	92.5 ± 5.9
Amplitude (mL·kg^−1^·min^−1^)	36.9 ± 1.8	36.59 ± 2.1	39.16 ± 2.0	38.5 ± 2.1
τ (s)	51.6 ± 2.6	52.3 ± 9.4	47.9 ± 2.2	49.6 ± 2.7
T50% (s)	55.1 ± 2.2	50.4 ± 3.4 *	53.9 ± 2.0	50.6 ± 1.5

EE$\dot{V}$O_2_ = End of exercise volume of oxygen; $\dot{V}$O_2max_ = maximum oxygen uptake; τ = time constant; T50% = 50% of end of exercise volume of oxygen. * denotes a significant within group difference (*p* = 0.026).

**Table 7:** Time to exhaustion, respiratory, exercise economy and perceived exertion parameters during maximal intensity exercise at visit 3 (pre-intervention) and 5 (post-intervention) for OliP and PL groups.

Variable	OliP		PL	
	Pre	Post	Pre	Post
TTE (s)	378.7 ± 13.5	393.1 ± 13.4 *	357.0 ± 15.7	377.5 ± 18.3 *
$\dot{V}$O_2max_ (mL·kg^−1^·min^−1^)	48.2 ± 2.6	48.0 ± 2.5	48.8 ± 2.6	50.8 ± 2.3 *
$\dot{V}$CO_2max_ (mL·kg^−1^·min^−1^)	53.4 ± 3.0	53.9 ± 2.8	52.7 ± 3.2	55.6 ± 3.2 *
$\dot{V}$_E_ (L·min^−1^)	141.29 ± 8.24	142.40 ± 8.86	136.76 ± 11.21	142.23 ± 10.70
RER	1.13 ± 0.01	1.14 ± 0.02	1.11 ± 0.02	1.13 ± 0.02
Speed at $\dot{V}$O_2max_ (km·h^−1^)	14.7 ± 0.7	14.9 ± 0.7	15.0 ± 0.9	15.4 ± 0.8
RPE	9.8 ± 0.1	9.6 ± 0.2	9.6 ± 0.1	9.8 ± 0.1

TTE = time to exhaustion; $\dot{V}$O_2max_ = maximal oxygen uptake; $\dot{V}$O_2_ = volume of oxygen; $\dot{V}$CO_2_ = volume of carbon dioxide; $\dot{V}$_E_ = minute ventilation; RER = respiratory exchange ratio; RPE = rating of perceived exertion. * denotes a significant within group difference (*p* ≤ 0.031).

## Data Availability

The data presented in this study is available upon request from the corresponding authors. The data is not publicly available due to ethical considerations, in accordance with participant consent on the use of confidential data.

## Supplementary Materials

The following supporting information can be downloaded at: https://www.mdpi.com/article/10.3390/nu15020421/s1, Table S1: List of food sources requested to be avoided by participants for the 3-days prior to main laboratory testing sessions; Table S2: Independent product analysis.

## References

[1] Hutchison A.T., Flieller E.B., Dillon K.J., Leverett B.D. (2014). Black Currant Nectar Reduces Muscle Damage and Inflammation Following a Bout of High-Intensity Eccentric Contractions. J. Diet. Suppl..

[2] Kelley D.S., Rasooly R., Jacob R.A., Kader A.A., Mackey B.E. (2006). Consumption of Bing Sweet Cherries Lowers Circulating Concentrations of Inflammation Markers in Healthy Men and Women. J. Nutr..

[3] Martín M.A., Ramos S. (2016). Cocoa polyphenols in oxidative stress: Potential health implications. J. Funct. Foods.

[4] Scalbert A., Williamson G. (2000). Dietary Intake and Bioavailability of Polyphenols. J. Nutr..

[5] Scalbert A., Morand C., Manach C., Rémésy C. (2002). Absorption and metabolism of polyphenols in the gut and impact on health. Biomed. Pharmacother..

[6] Tsao R. (2010). Chemistry and Biochemistry of Dietary Polyphenols. Nutrients.

[7] Cory H., Passarelli S., Szeto J., Tamez M., Mattei J. (2018). The Role of Polyphenols in Human Health and Food Systems: A Mini-Review. Front. Nutr..

[8] Heydenreich J., Kayser B., Schutz Y., Melzer K. (2017). Total Energy Expenditure, Energy Intake, and Body Composition in Endurance Athletes Across the Training Season: A Systematic Review. Sports Med. -Open.

[9] Abbiss C.R., Laursen P.B. (2005). Models to Explain Fatigue during Prolonged Endurance Cycling. Sports Med..

[10] Hausswirth C., Le Meur Y. (2011). Physiological and nutritional aspects of post-exercise recovery: Specific recommendations for female athletes. Sports Med..

[11] D’Angelo S. (2019). Polyphenols and Athletic Performance: A Review on Human Data. Plant Physiological Aspects of Phenolic Compounds.

[12] Finkel T. (2011). Signal transduction by reactive oxygen species. J. Cell Biol..

[13] Gomez-Cabrera M.-C., Domenech E., Viña J. (2008). Moderate exercise is an antioxidant: Upregulation of antioxidant genes by training. Free. Radic. Biol. Med..

[14] Margaritelis N.V., Theodorou A.A., Paschalis V., Veskoukis A.S., Dipla K., Zafeiridis A., Panayiotou G., Vrabas I.S., Kyparos A., Nikolaidis M.G. (2017). Adaptations to endurance training depend on exercise-induced oxidative stress: Exploiting redox interindividual variability. Acta Physiol..

[15] Tryfidou D.V., McClean C., Nikolaidis M.G., Davison G.W. (2020). DNA Damage Following Acute Aerobic Exercise: A Systematic Review and Meta-analysis. Sports Med..

[16] Donato A.J., Uberoi A., Bailey D.M., Wray D.W., Richardson R.S. (2010). Exercise-induced brachial artery vasodilation: Effects of antioxidants and exercise training in elderly men. Am. J. Physiol. Circ. Physiol..

[17] Powers S.K., Talbert E.E., Adhihetty P.J. (2011). Reactive oxygen and nitrogen species as intracellular signals in skeletal muscle. J. Physiol..

[18] Perkins I.C., Vine S.A., Blacker S.D., Willems M. (2015). New Zealand Blackcurrant Extract Improves High-Intensity Intermittent Running. Int. J. Sport Nutr. Exerc. Metab..

[19] Deley G., Guillemet D., Allaert F., Babault N. (2017). An Acute Dose of Specific Grape and Apple Polyphenols Improves Endurance Performance: A Randomized, Crossover, Double-Blind versus Placebo Controlled Study. Nutrients.

[20] Fisher N.D.L., Hughes M., Gerhard-Herman M., Hollenberg N.K. (2003). Flavanol-rich cocoa induces nitric-oxide-dependent vasodilation in healthy humans. J. Hypertens..

[21] Davis J.M., Carlstedt C.J., Chen S., Carmichael M.D., Murphy E.A. (2010). The Dietary Flavonoid Quercetin Increases VO2max and Endurance Capacity. Int. J. Sport Nutr. Exerc. Metab..

[22] Davis J.M., Murphy E.A., Carmichael M.D., Davis B. (2009). Quercetin increases brain and muscle mitochondrial biogenesis and exercise tolerance. Am. J. Physiol. Integr. Comp. Physiol..

[23] Westphal C., Dipp M., Guarente L. (2007). A therapeutic role for sirtuins in diseases of aging?. Trends Biochem. Sci..

[24] Chung S., Yao H., Caito S., Hwang J.-W., Arunachalam G., Rahman I. (2010). Regulation of SIRT1 in cellular functions: Role of polyphenols. Arch. Biochem. Biophys..

[25] Kamei Y., Miura S., Suzuki M., Kai Y., Mizukami J., Taniguchi T., Mochida K., Hata T., Matsuda J., Aburatani H. (2004). Skeletal Muscle FOXO1 (FKHR) Transgenic Mice Have Less Skeletal Muscle Mass, Down-regulated Type I (Slow Twitch/Red Muscle) Fiber Genes, and Impaired Glycemic Control. J. Biol. Chem..

[26] Lu H., Huang H. (2011). FOXO1: A potential target for human diseases. Curr. Drug Targets.

[27] Lagouge M., Argmann C., Gerhart-Hines Z., Meziane H., Lerin C., Daussin F., Messadeq N., Milne J., Lambert P., Elliott P. (2006). Resveratrol improves mitochondrial function and protects against metabolic disease by activating SIRT1 and PGC-1α. Cell.

[28] Wood dos Santos T., Cristina Pereira Q., Teixeira L., Gambero A., Villena J.A., Lima Ribeiro M. (2018). Effects of polyphenols on thermogenesis and mitochondrial biogenesis. Int. J. Mol. Sci..

[29] Bowtell J., Kelly V. (2019). Fruit-Derived Polyphenol Supplementation for Athlete Recovery and Performance. Sports Med..

[30] Myburgh K.H. (2014). Polyphenol supplementation: Benefits for exercise performance or oxidative stress?. Sports Med..

[31] Bowtell J.L., Sumners D.P., Dyer A., Fox P., Mileva K.N. (2011). Montmorency Cherry Juice Reduces Muscle Damage Caused by Intensive Strength Exercise. Med. Sci. Sports Exerc..

[32] Connolly D., McHugh M., Padilla-Zakour O. (2006). Efficacy of a tart cherry juice blend in preventing the symptoms of muscle damage. Brit. J. Sports Med..

[33] Howatson G., McHugh M.P., Hill J.A., Brouner J., Jewell A.P., Van Someren K.A., Shave R.E., Howatson S.A. (2010). Influence of tart cherry juice on indices of recovery following marathon running. Scand. J. Med. Sci. Sports.

[34] Rietjens S.J., Bast A., de Vente J., Haenen G.R.M.M. (2007). The olive oil antioxidant hydroxytyrosol efficiently protects against the oxidative stress-induced impairment of the NO^•^ response of isolated rat aorta. Am. J. Physiol. Circ. Physiol..

[35] Rietjens S.J., Bast A., Haenen G.R.M.M. (2007). New Insights into Controversies on the Antioxidant Potential of the Olive Oil Antioxidant Hydroxytyrosol. J. Agric. Food Chem..

[36] Covas M.-I., Nyyssönen K., Poulsen H.E., Kaikkonen J., Zunft H.-J.F., Kiesewetter H., Gaddi A., de la Torre R., Mursu J., Bäumler H. (2006). The effect of polyphenols in olive oil on heart disease risk factors: A randomized trial. Ann. Intern. Med..

[37] Marrugat J., Covas M.-I., Fitó M., Schroder H., Miró-Casas E., Gimeno E., López-Sabater M.C., de la Torre R., Farré M., the members of the SOLOS Investigators (2004). Effects of differing phenolic content in dietary olive oils on lipids and LDL oxidation. Eur. J. Nutr..

[38] Weinbrenner T., Fitó M., de la Torre R., Saez G.T., Rijken P., Tormos C., Coolen S., Albaladejo M.F., Abanades S., Schroder H. (2004). Olive Oils High in Phenolic Compounds Modulate Oxidative/Antioxidative Status in Men. J. Nutr..

[39] Manna C., Galletti P., Cucciolla V., Moltedo O., Leone A., Zappia V. (1997). The protective effect of the olive oil polyphenol (3,4-Dihydroxyphenyl)-ethanol counteracts reactive oxygen metabolite–induced cytotoxicity in Caco-2 cells. J. Nutr..

[40] Goya L., Mateos R., Bravo L. (2007). Effect of the olive oil phenol hydroxytyrosol on human hepatoma HepG2 cells. Eur. J. Nutr..

[41] Faul F., Erdfelder E., Lang A.-G., Buchner A. (2007). G* Power 3: A flexible statistical power analysis program for the social, behavioral, and biomedical sciences. Behav. Res. Methods.

[42] Riva A., Vitale J.A., Belcaro G., Hu S., Feragalli B., Vinciguerra G., Cacchio M., Bonanni E., Giacomelli L., Eggenhöffner R. (2018). Quercetin phytosome^®^ in triathlon athletes: A pilot registry study. Minerva Med..

[43] Hopkins W.G., Schabort E.J., Hawley J.A. (2001). Reliability of Power in Physical Performance Tests. Sports Med..

[44] Ward S.A. (2018). Open-circuit respirometry: Real-time, laboratory-based systems. Eur. J. Appl. Physiol..

[45] Roberts J.D., Lillis J., Pinto J.M., Willmott A.G.B., Gautam L., Davies C., López-Samanes Á., Del Coso J., Chichger H. (2022). The Impact of a Natural Olive-Derived Phytocomplex (OliPhenolia^®^) on Exercise-Induced Oxidative Stress in Healthy Adults. Nutrients.

[46] James C., Richardson A., Watt P.W., Willmott A., Gibson O.R., Maxwell N.S. (2017). Short-term heat acclimation improves the determinants of endurance performance and 5-km running performance in the heat. Appl. Physiol. Nutr. Metab..

[47] Longman D.P., Merzbach V., Pinto J.M., Atkinson L.H., Wells J.C.K., Gordon D., Stock J.T. (2022). Alternative Metabolic Strategies are Employed by Endurance Runners of Different Body Sizes; Implications for Human Evolution. Adapt. Hum. Behav. Physiol..

[48] Jones A.M., Doust J.H. (1996). A 1% treadmill grade most accurately reflects the energetic cost of outdoor running. J. Sports Sci..

[49] Bentley D.J., Newell J., Bishop D. (2007). Incremental Exercise Test Design and Analysis. Sports Med..

[50] Faude O., Kindermann W., Meyer T. (2009). Lactate Threshold Concepts. Sports Med..

[51] Winter E.M., Jones A.M., Davison R.R., Bromley P.D., Mercer T.H. (2006). Sport and Exercise Physiology Testing Guidelines: Volume I–Sport Testing: The British Association of Sport and Exercise Sciences Guide.

[52] Zacca R., Azevedo R., Figueiredo P., Vilas-Boas J.P., Castro F.A.D.S., Pyne D.B., Fernandes R.J. (2019). VO2FITTING: A Free and Open-Source Software for Modelling Oxygen Uptake Kinetics in Swimming and other Exercise Modalities. Sports.

[53] Jones A.M., Poole D.C. (2005). Oxygen Uptake Dynamics: From Muscle to Mouth—An Introduction to the Symposium. Med. Sci. Sports Exerc..

[54] Robergs R.A., Dwyer D., Astorino T. (2010). Recommendations for Improved Data Processing from Expired Gas Analysis Indirect Calorimetry. Sports Med..

[55] Roberts J., Willmott A., Beasley L., Boal M., Davies R., Martin L., Chichger H., Gautam L., Del Coso J. (2021). The Impact of Decaffeinated Green Tea Extract on Fat Oxidation, Body Composition and Cardio-Metabolic Health in Overweight, Recreationally Active Individuals. Nutrients.

[56] Shapiro S.S., Wilk M.B., Chen H.J. (1968). A comparative study of various tests for normality. J. Am. Stat. Assoc..

[57] Bloomer R.J., Goldfarb A.H., Wideman L., Mckenzie M.J., Consitt L.A. (2005). Effects of acute aerobic and anaerobic exercise on blood markers of oxidative stress. J. Strength Cond. Res..

[58] Bloomer R.J., Goldfarb A.H., McKenzie M.J. (2006). Oxidative stress response to aerobic exercise: Comparison of antioxidant supplements. Med. Sci. Sport. Exerc..

[59] Feng Z., Bai L., Yan J., Li Y., Shen W., Wang Y., Wertz K., Weber P., Zhang Y., Chen Y. (2011). Mitochondrial dynamic remodeling in strenuous exercise-induced muscle and mitochondrial dysfunction: Regulatory effects of hydroxytyrosol. Free Radic. Biol. Med..

[60] Friedel A., Raederstorff D., Roos F., Toepfer C., Wertz K. (2013). Hydroxytyrosol Benefits Muscle Differentiation and Muscle Contraction and Relaxation. U.S. Patent.

[61] Hao J., Shen W., Yu G., Jia H., Liu J., Feng Z., Wang Y., Weber P., Wertz K., Sharman E. (2010). Hydroxytyrosol promotes mitochondrial biogenesis and mitochondrial function in 3T3-L1 adipocytes. J. Nutr. Biochem..

[62] Signorile A., Micelli L., De Rasmo D., Santeramo A., Papa F., Ficarella R., Gattoni G., Scacco S., Papa S. (2014). Regulation of the biogenesis of OXPHOS complexes in cell transition from replicating to quiescent state: Involvement of PKA and effect of hydroxytyrosol. BBA-Mol. Cell Res..

[63] Leri M., Scuto M., Ontario M.L., Calabrese V., Calabrese E.J., Bucciantini M., Stefani M. (2020). Healthy Effects of Plant Polyphenols: Molecular Mechanisms. Int. J. Mol. Sci..

[64] Al Fazazi S., Casuso R.A., Aragón-Vela J., Casals C., Huertas J.R. (2018). Effects of hydroxytyrosol dose on the redox status of exercised rats: The role of hydroxytyrosol in exercise performance. J. Int. Soc. Sports Nutr..

[65] Casuso R.A., Al-Fazazi S., Hidalgo-Gutierrez A., López L.C., Plaza-Díaz J., Rueda-Robles A., Huertas J.R. (2019). Hydroxytyrosol influences exercise-induced mitochondrial respiratory complex assembly into supercomplexes in rats. Free Radical Bio. Med..

[66] Simioni C., Zauli G., Martelli A.M., Vitale M., Sacchetti G., Gonelli A., Neri L.M. (2018). Oxidative stress: Role of physical exercise and antioxidant nutraceuticals in adulthood and aging. Oncotarget.

[67] Bailey S.J., Wilkerson D.P., DiMenna F.J., Jones A.M. (2009). Influence of repeated sprint training on pulmonary O_2_ uptake and muscle deoxygenation kinetics in humans. J. Appl. Physiol..

[68] Breese B.C., McNarry M.A., Marwood S., Blackwell J.R., Bailey S.J., Jones A.M. (2013). Beetroot juice supplementation speeds O_2_uptake kinetics and improves exercise tolerance during severe-intensity exercise initiated from an elevated metabolic rate. Am. J. Physiol. Integr. Comp. Physiol..

[69] Christensen P.M., Jacobs R.A., Bonne T.C., Flück D., Bangsbo J., Lundby C. (2016). A short period of high-intensity interval training improves skeletal muscle mitochondrial function and pulmonary oxygen uptake kinetics. J. Appl. Physiol..

[70] Navarro A., Boveris A. (2007). The mitochondrial energy transduction system and the aging process. Am. J. Physiol. Physiol..

[71] Zheng A., Li H., Xu J., Cao K., Li H., Pu W., Yang Z., Peng Y., Long J., Liu J. (2015). Hydroxytyrosol improves mitochondrial function and reduces oxidative stress in the brain of *db/db* mice: Role of AMP-activated protein kinase activation. Br. J. Nutr..

[72] Cogliati S., Enriquez J.A., Scorrano L. (2016). Mitochondrial Cristae: Where Beauty Meets Functionality. Trends Biochem. Sci..

[73] Whitfield J., Ludzki A., Heigenhauser G.J.F., Senden J.M.G., Verdijk L.B., van Loon L.J.C., Spriet L.L., Holloway G.P. (2015). Beetroot juice supplementation reduces whole body oxygen consumption but does not improve indices of mitochondrial efficiency in human skeletal muscle. J. Physiol..

[74] Bailey S.J., Winyard P., Vanhatalo A., Blackwell J.R., DiMenna F.J., Wilkerson D.P., Tarr J., Benjamin N., Jones A.M. (2009). Dietary nitrate supplementation reduces the O_2_ cost of low-intensity exercise and enhances tolerance to high-intensity exercise in humans. J. Appl. Physiol..

[75] Liu F., Wanigatunga A.A., Zampino M., Knuth N.D., Simonsick E.M., Schrack J.A., Ferrucci L. (2020). Association of Mitochondrial Function, Substrate Utilization, and Anaerobic Metabolism With Age-Related Perceived Fatigability. J. Gerontol. Ser. A.

[76] Zhu L., Liu Z., Feng Z., Hao J., Shen W., Li X., Sun L., Sharman E., Wang Y., Wertz K. (2010). Hydroxytyrosol protects against oxidative damage by simultaneous activation of mitochondrial biogenesis and phase II detoxifying enzyme systems in retinal pigment epithelial cells. J. Nutr. Biochem..

[77] Ditano-Vázquez P., Torres-Peña J.D., Galeano-Valle F., Pérez-Caballero A.I., Demelo-Rodríguez P., Lopez-Miranda J., Katsiki N., Delgado-Lista J., Alvarez-Sala-Walther L.A. (2019). The Fluid Aspect of the Mediterranean Diet in the Prevention and Management of Cardiovascular Disease and Diabetes: The Role of Polyphenol Content in Moderate Consumption of Wine and Olive Oil. Nutrients.

[78] vila-Román J., Soliz-Rueda J.R., Bravo F.I., Aragonès G., Suárez M., Arola-Arnal A., Mulero M., Salvadó M.-J., Arola L., Torres-Fuentes C. (2021). Phenolic compounds and biological rhythms: Who takes the lead?. Trends Food Sci. Tech..

[79] Corona G., Tzounis X., Dessì M.A., Deiana M., Debnam E.S., Visioli F., Spencer J.P.E. (2006). The fate of olive oil polyphenols in the gastrointestinal tract: Implications of gastric and colonic microflora-dependent biotransformation. Free Radic. Res..

[80] Caruso D., Visioli F., Patelli R., Galli C., Galli G. (2001). Urinary excretion of olive oil phenols and their metabolites in humans. Metabolism.

[81] Castillo-Luna A., Ledesma-Escobar C., Gómez-Díaz R., Priego-Capote F. (2022). The secoiridoid profile of virgin olive oil conditions phenolic metabolism. Food Chem..

[82] Ricelli A., Gionfra F., Percario Z., De Angelis M., Primitivo L., Bonfantini V., Antonioletti R., Bullitta S.M., Saso L., Incerpi S. (2020). Antioxidant and Biological Activities of Hydroxytyrosol and Homovanillic Alcohol Obtained from Olive Mill Wastewaters of Extra-Virgin Olive Oil Production. J. Agric. Food Chem..

[83] Lee H.C., Jenner A.M., Low C.S., Lee Y.K. (2006). Effect of tea phenolics and their aromatic fecal bacterial metabolites on intestinal microbiota. Res. Microbiol..

[84] Morgan D.W., Martin P.E., Krahenbuhl G.S. (1989). Factors Affecting Running Economy. Sports Med..

[85] Saunders P.U., Pyne D.B., Telford R.D., Hawley J.A. (2004). Factors Affecting Running Economy in Trained Distance Runners. Sports Med..

[86] Jacobs R.A., Lundby C. (2013). Mitochondria express enhanced quality as well as quantity in association with aerobic fitness across recreationally active individuals up to elite athletes. J. Appl. Physiol..

[87] Menendez J.A., Joven J., Aragonès G., Barrajón-Catalán E., Beltrán-Debón R., Borrás-Linares I., Camps J., Corominas-Faja B., Cufí S., Fernández-Arroyo S. (2013). Xenohormetic and anti-aging activity of secoiridoid polyphenols present in extra virgin olive oil: A new family of gerosuppressant agents. Cell Cycle.

[88] Enríquez J.A. (2016). Supramolecular Organization of Respiratory Complexes. Annu. Rev. Physiol..

[89] Greggio C., Jha P., Kulkarni S.S., Lagarrigue S., Broskey N.T., Boutant M., Wang X., Alonso S.C., Ofori E., Auwerx J. (2016). Enhanced Respiratory Chain Supercomplex Formation in Response to Exercise in Human Skeletal Muscle. Cell Metab..

[90] Rasmussen P., Nielsen J., Overgaard M., Krogh-Madsen R., Gjedde A., Secher N.H., Petersen N.C. (2010). Reduced muscle activation during exercise related to brain oxygenation and metabolism in humans. J. Physiol..

[91] Maughan R.J., Burke L.M., Dvorak J., Larson-Meyer D.E., Peeling P., Phillips S.M., Rawson E.S., Walsh N.P., Garthe I., Geyer H. (2018). IOC Consensus Statement: Dietary Supplements and the High-Performance Athlete. Int. J. Sport Nutr. Exerc. Metab..

[92] Rickards L., Lynn A., Barker M.E., Russell M., Ranchordas M.K. (2022). Comparison of the polyphenol content and in vitro antioxidant capacity of fruit-based nutritional supplements commonly consumed by athletic and recreationally active populations. J. Int. Soc. Sports Nutr..

