# Supplementary material for: The Effect of a Hydroxytyrosol-Rich, Olive-Derived Phytocomplex on Aerobic Exercise and Acute Recovery
Source: Nutrients. 2023 Jan 13;15(2):421. doi: 10.3390/nu15020421 (PMC9864860; doi:10.3390/nu15020421)
Supplement: Supplementary file 1 [file nutrients-15-00421-s001.zip › nutrients-2129851-supplementary.pdf]

Supplementary Material

Table S1: List of food sources requested to be avoided by participants for the 3-days prior to main laboratory testing sessions.

Individual food items were cross referenced against the U.S. Department of Agriculture (USDA) database on the flavonoid content of selected foods, and the Phenol-Explorer database. Total polyphenol content of food source presented for reference in mg·100 mL^{-1} or mg·$100g^{-1}$ as applicable.

Food source	Polyphenol content
Wine - Grape wines (Red, Rosé, White)	215.5, 82.2, 32.1
Alcohol – Beer Ale and Cider	41.6, 52.3, 98.3
Oils: Virgin olive oil, Extra virgin Olive oil, refined	20.7, 55.1, 19.8
Olives: Black, Green	117.2, 161.2
Caffeine: Coffee, Tea	266.7
Turmeric	2117.0
Beetroot and beetroot juice	164.1
Dark chocolate	1859.9
Pecans	1284.0
Blueberries	471.6
Strawberries	289.20
Dried berries: Cranberries, raisins, chokeberries, blackcurrants, (all) *	915.0
Goji Berries	31.2
Raspberries	154.7
Figs	960.0
Grapes: Red, Green	185.0, 121.8
Kale	176.7
Red cabbage	119.9
Spinach	248.1
Artichokes	1142.4

* = Mean of primary groups.

Table S2 - Independent product analysis

Independent laboratory analysis was undertaken on samples of both products (Analytical Group SRL, Florence, Italy) using an internal method based on the determination of biophenols according to the International Olive Council method (see COI/T.20/Doc n 29 rev 1 2017) using liquid chromatography with a detector wavelength of 280 nm (with results expressed in g of Tyrosol·L^{-1} water using an internal standard). Overall findings are shown below and converted to provide estimated analyses per 28 mL serve.

	**OliP (mg·L^{-1})**	**PL (mg·L^{-1})**	**OliP (/28 mL serve)**	**PL (/28 mL serve)**
Total Phenolic Profile	11282	1748	315.9	48.9
Hydroxytyrosol	1030	0	28.8	0
3-4-DHPEA-EDA	810	0	22.7	0
Verbascoid	570	0	16.0	0
Oleuropein aglycone	310	0	8.7	0
Hydroxytyrosol glucoside	290	0	8.1	0
Tyrosol glycosylated derivatives	240	0	6.7	0
Hydroxy-verbascoid isomer 2	220	0	6.2	0
Hydroxy-verbascoid isomer 1	200	0	5.6	0
p-coumaroyl secologanoside	190	0	5.3	0
Isoverbascoid	0	0	0	0
Rutin	0	0	0	0
Chlorogenic acid	0	0	0	0
Caffeic acid	0	0	0	0
Luteolin-7-O-Glucoside	0	0	0	0
Nuzhenide	0	0	0	0
p-HPEA-EDA	0	0	0	0
Caffeoyl secologanoside	0	0	0	0
